# A Case of Cut Throat

**Published:** 1889-01

**Authors:** J. M. Taylor

**Affiliations:** Corinth, Miss


### A CASE OF CUT THROAT.*

BY J. M. TAYLOR, M. D., OF CORINTH, MISS.

On the 29th day of October, 1888, Robert Watson, about 24 years of age, under an insane impulse, cut his throat with a dull pocket-knife. The wound was made transversely through the thyro-hyoid membrane, separating the larynx from the base of the tongue and opening the pharynx. The depressor muscles of the hyoid bone, the sterno-hyoid, the thyro-hyoid and the omo-hyoid, I think were all severed. The larynx and trachea dropped down, leaving a gaping wound five inches long and two inches wide. The carotid arteries could be seen plainly pulsating in either angle of the wound. The larynx and trachea thus set free from attachment above, and acted on by the sterno-thyroid muscles, projected forwards and afforded a beautiful view of the inside of the vocal box in the living subject. The finger passed through the wound, readily entered the pharynx and fauces, or passed through the mouth and around the base of the tongue, if long enough, would have come out at the wound. Water taken into the mouth all ran out of the wound. All power of deglutition was lost. The wound was inflicted in Tishomingo county, Mississippi, at a sawmill ten miles south of Iuka. I was

*Read before the Southern Sur[illegible] and Gynecological Association.

called to see the patient with Drs. R. S. and W. A. Hodges, and arrived there, over forty miles, about twenty hours after the occurrence. At that time all hemorrhage had ceased; it had been quite free but not excessive. The patient was resting very quietly and breathing very easy through the yawning wound. Pulse fairly good and mind apparently entirely clear. The patient is a young man of exemplary character, exceptionally good morals, well educated, a Methodist minister, and son of the proprietor of the mill. Coaptation of the wounded structures in the deeper parts of the wound was impracticable, but we drew the skin and fascia together and closed the external wound with eight or ten silk stitches. The edges of the wound came together so accurately that the line was scarcely visible, and neither blood, mucous, nor air escaped through it. Breathing through the nose and mouth was perfectly free and easy—entirely normal. I hoped for healing of the external wound by first intention, and I believe it would have occurred if the head could have been kept bent forwards. But we were compelled to support him by means of the stomach tube, the frequent introduction of which caused the stitches to yield so that air and mucous passed between the edges of wound. To introduce the tube it was necessary to tilt the head strongly backwards, and the finger in the fauces to guide the tube caused efforts to vomit. Notwithstanding all this there was but little leakage for four or five days. But after that time, slight suppuration along the suture tracts caused them to yield and the wound began to gape and some of the stitches cut through. We removed the balance as they were doing no good. The wound had closed only for a short distance in each angle. We attempted to approximate the lips of the wound with plasters, but the beard and the sharp angle under the jaw interfered with the adhesion of the plasters, and the frequent bending of the neck back to introduce the stomach tube made them very inefficient. Healthy granulations appeared on all the wounded surfaces, and the patient got along much better than we expected. He took one pint of milk with an egg and a little sugar beat up in it twice a day, through the stomach tube. He rested well most of the time. A few times he manifested some mental aberration but chloral and bromide of potassium by enema acted admirably. This, with a few hypodermic doses of morphine, and some laxative enemata, constituted all the medication required. He desired to recover and co-operated cheerfully in everything done for him. He became very tired of the stomach tube and Dr. W. A. Hodges, who had the immediate charge of the case, tried nutritive enemata, but after five days it was deemed best to return to the stomach tube. The egg being a little heavy on the stomach was left off and he got along very well on one and a half pints of milk twice a day. He retained his strength well and did not emaciate perceptibly. Just one month after the injury was done he recovered the power of swallowing liquids, and now (December 4th) the opening in the larynx is less than a quarter of an inch in diameter and the external wound less than one and a half inches. He is all the time hungry and is gaining strength every day. The simple water dressing at first and a little cosmoline or cerate afterwards kept the wound in as fine condition as could be. There was scarcely any suppuration at any time. No antiseptics whatever were used. There was no cough, or other symptom indicating laryngitis or bronchitis.

*Read before the Southern Surgical and Gynecological Association.

